# The Geographic Movement Patterns and Career and Personal Interests of Orthopaedic Surgery Residents in the United States

**DOI:** 10.5435/JAAOSGlobal-D-21-00056

**Published:** 2021-09-02

**Authors:** Nathan C. Sherman, Jacob C. Sorenson, Ansab M. Khwaja, Gregory L. DeSilva

**Affiliations:** From the Department of Orthopaedic Surgery, University of Arizona (Dr. Sherman, Dr. Khwaja, Dr. DeSilva), and College of Medicine (Sorenson), University of Arizona, Tucson, AZ.

## Abstract

Orthopaedic surgery continues to be a highly desired residency specialty for graduating medical students in the United States. The geographic preferences and trajectories of orthopaedic surgery applicants are not well understood.

**Objective:**

The primary objective of this study was to determine the geographic movement patterns of orthopaedic residents from university through residency. A secondary objective was to trend the career and personal interests of orthopaedic residents.

**Methods:**

One hundred eighty-seven orthopaedic surgery programs and 3672 residents were identified through the Electronic Residency Application Service website and Google searches and included for study. Program provided information, including the residents' medical school, undergraduate institution, career interests, and personal interests was then gathered. All data were recorded between the dates of March 25, 2020, and April 2, 2020.

**Results:**

A minority of orthopaedic residents (46.2%; n = 1,569/3,398) were training in the same geographic region of their medical school; however, analysis revealed a statistically significant strength of association for home region over a different US census bureau region (χ^2^ = 127.4, df = 8, Cramer's V = 0.2, *P* < 0.001). The average distance between orthopaedic residents' medical school and residency program was 666 miles. Male residents were more interested in arthroplasty, spine, and sports, whereas female residents were more interested in hand and pediatrics. The residents leading interests were in arthroplasty (24.4%), sports (21.7%), and trauma (21.3%).

**Conclusion:**

Orthopaedic surgery residents are more likely to train in a geographical region that is different from their medical school or undergraduate institution. The reported career interests of male and female orthopaedic residents showed significant differences, but personal interests seem to be similar between genders.

Orthopaedic surgery continues to be a highly desired residency specialty for graduating medical students in the United States. Selection criteria for orthopaedic surgery residents have been well reported,^1,2,3,4^ and academic criteria such as US Medical Licensing Examination step 1 and 2 scores, Alpha Omega Alpha status, and class rank are frequently cited as factors contributing to higher ranking and match probability. Quality of the interview, letters of recommendation, and research are also major considerations. Prospective applicants' geographic preferences, however, may also have a substantial effect in the ranking and match process.^5,6^ One study found that geographic location was the most significant factor affecting prospective resident decision making in ranking a residency program.^7^

More than half of the otolaryngology applicants (58%) and 48% of plastic surgery applicants match in the same geographic region for residency in which they attended medical school.^8,9^ Ophthalmology applicants have a 60% probability of matching in the same geographic region.^10^ Using data from the 2015 to 2016 academic year, Cox et al.^11^ were the first to discuss orthopaedic resident geography trends and reported that 47% of medical students remained in the same geographic region for their orthopaedic residency program. The National Resident Matching Program (NRMP) Match data from 2018 demonstrated that 63% of all residency applicants matched in their home geographic region.^12^ Each of these studies collectively indicates that many applicants will remain in the same geographic location for residency as they did for medical school; however, the reasons for this trend are unclear.

The previous study on orthopaedic resident geography did not take note of physical distance between programs. In addition, few data have been reported on the career or personal interests of orthopaedic residents. Away rotations are an invaluable opportunity for medical students to learn the culture of different programs and to identify whether their career and personal interests align with the cultures of those programs. Discussions with current residents during away rotations have been demonstrated to be a major influence in medical students' rank lists for orthopaedic surgery programs.^13^ On average, a medical student will perform 2.4 away rotations and spend $2,799 on rotations alone,^14^ but medical students may have difficulty rotating at each program in which they have interest. Standardized and easily accessible online resident profiles may allow medical students to assess whether their career and personal interests align with current residents.

The primary objective of this study was to identify and update the current geographic trajectories of orthopaedic residents from university through residency. This study aims to assist both orthopaedic surgery residency programs and prospective residents by describing the geographic paths of medical students matching into residency, which may affect the application and interview decision-making process. A secondary objective was to trend the career and personal interests of orthopaedic residents and assess for gender differences. It was hypothesized that orthopaedic residents are more likely to train at programs within the same geographic regions as their universities and medical schools.

## Methods

Existing orthopaedic surgery residency programs were identified on the Electronic Residency Application Service (ERAS) website, a methodology adapted from previous studies of similar focus.^8,11^ Orthopaedic residency program websites were identified through ERAS listings and search engine queries through Google. If an ERAS listing did not include a direct link to a program's website, a Google search was performed by the authors (N.S. or J.S.) using search queries including the name of the specific program's orthopaedic surgery website. Only information provided by the institutional program websites was included, and any program not participating in the 2019 to 2020 Match was excluded from study. No inquiries were made by the study team for resident information, and all data were self-reported by the program. No collateral sources were used, and any link outside of the specific program's website was excluded. The available information provided by the institutional website, including the residents' medical school, undergraduate institution, career interests, and life interests, was then gathered and coded. All data were recorded between 03/25/2020 and 04/02/2020.

Each resident was coded into one of nine specific geographic regions for their residency program, medical school, and undergraduate institution based on the US Census Bureau state divisions.^15^ In addition to specific geographic regions, institutional addresses were found through search engine query of that institution's name and compared between a resident's orthopaedic program and home medical school and the orthopaedic program and undergraduate institution. This approach allowed for a precise mileage to determine the average distance an orthopaedic surgery resident traveled to attend a particular program. This distance was calculated using the Virtual Basic for Applications Function in Excel, which used the Google Maps Application Program Interface Distance Matrix Function to produce the distance in miles.

Data for career interests were compiled into 10 different categories, including arthroplasty/reconstruction, foot and ankle, hand, oncology, pediatrics, shoulder and elbow, spine, sports, trauma, and undecided/other. Data were coded as “0” for no indicated interest or “1” for interested, based on the presence of these criteria in each resident's profile. Data for lifestyle interests were compiled into seven different categories, including cooking, exercise, family, outdoor activities/sports, travel, video games/movie/entertainment, and other. Data were also coded as “0” for no indicated interest or “1” for interested, based on the presence of these criteria in each resident's profile.

Chi-square analyses were performed, and the Phi correlation coefficient and Cramer's V were calculated for the strength of association. *P* values were then calculated to determine the significance of association. All data analyses were performed using Microsoft Excel, version 16.35, and IBM SPSS Data Analysis Software (IBM Corp. Released 2017. IBM SPSS Statistics for Windows, Version 26.0.).

This research did not receive any specific grant from funding agencies in the public, commercial, or not-for-profit sectors. All information used in this study was freely available on orthopaedic residency program websites, and institutional review board exempt approval was granted.

## Results

The ERAS website listed 189 orthopaedic surgery residency programs. Of these programs, two did not participate in the match for the 2019 to 2020 cycle and were excluded from analysis, leaving 187 programs. At the time of study, 4,138 residents were being trained in these programs, although only 3,672 residents were identified based on program-provided information present in the program websites. Of the 3,672 residents included in the study, programs reported medical schools for 3,398 (92.5%) and universities for 1741 (47.4%) residents.

**Figure 1:**
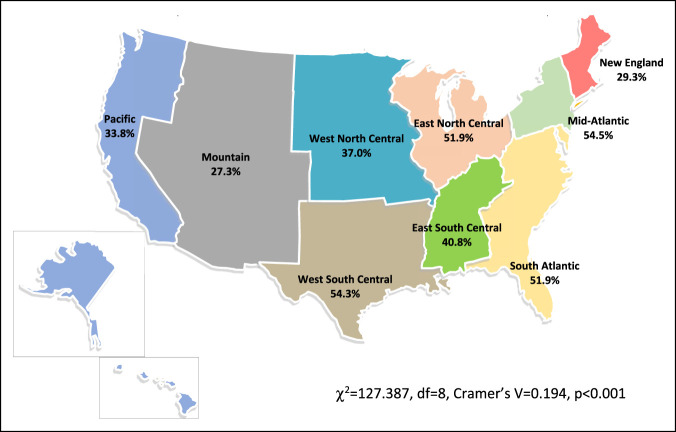
Map showing residents in the same region as their medical school.

**Figure 2:**
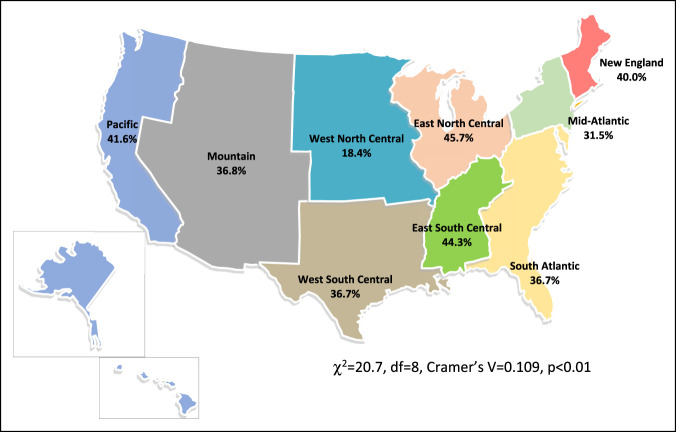
Map showing residents in the same region as their undergraduate institution.

A minority of orthopaedic residents (46.2%; n = 1,569/3,398) were training in the same geographic region of their medical school; however, analysis revealed a statistically significant strength of association for home region over a different US census bureau region (χ^2^ = 127.4, df = 8, Cramer's V = 0.2, *P* < 0.001) (Table 1, Figure 1). Similarly, residents had a statistically significant probability (697, 40.0%) of attending university and residency (χ^2^ = 20.7, df = 8, Cramer's V = 0.1, *P* < 0.01) (Table 1, Figure 2) and university and medical school (959, 55.1%) (χ^2^ = 43.6, df = 8, Cramer's V = 0.2, *P* < 0.001) (Table 1) in the same geographic region. The average distance between orthopaedic residents' medical school and residency program was 666 miles (SD = 789), with a maximum of 3331 miles. The mean distance between undergraduate institution and residency program was 779 miles (SD = 821), with a maximum distance of 3157 miles.

**Table 1:** Percentage of Residents in the Same/Different Census Region as Their Medical School Location

US Census Division, No. of Programs	Same Region, No. of Residents (%)	Different Region, No. of Residents (%)	Total, No.
Pacific—20	141 (33.8%)	276 (66.2%)	417
Mountain—8	41 (27.3%)	109 (72.7%)	150
West North Central—12	88 (37.0%)	150 (63.0%)	238
East North Central—42	382 (51.9%)	354 (48.1%)	736
West South Central—19	145 (54.3%)	122 (45.7%)	267
East South Central—10	53 (40.3%)	77 (59.2%)	130
South Atlantic—26	280 (51.9%)	260 (48.1%)	540
Middle Atlantic—40	367 (54.5%)	307 (45.5%)	674
New England—11	72 (29.3%)	174 (70.7%)	246
Total—188	1,569 (46.2%)	1,829 (53.8%)	3398

χ^2^ = 127.387, df = 8, Cramer's V = 0.194, *P* < 0.001.

Residency programs inconsistently reported resident research (3.9%) and other advanced degrees obtained by residents (2.9%). Only 607 (16.5%) orthopaedic residents had career interests listed by the residency program (Table 2). Of the specialty interests provided, most residents were interested in arthroplasty (148, 24.4%), followed by sports (132, 21.7%), trauma (129, 21.3%), hand (97, 16.0%), spine (79, 13.0%), and shoulder and elbow (74, 12.2%). Of the sample, residents were least interested in pediatrics (35, 5.8%), foot and ankle (27, 4.4%), and oncology (23, 3.8%). Of note, 180 (29.7%) residents reported that they were undecided about their specialty interests.

**Table 2:** Percentage of Residents in the Same/Different Census Region as Their Undergraduate Institution Location

US Census Division, No. of Programs	Same Region, No. of Residents (%)	Different Region, No. of Residents (%)	Total, No.
Pacific—20	109 (41.6%)	153 (58.4%)	262
Mountain—8	32 (36.8%)	55 (63.2%)	87
West North Central—12	7 (18.4%)	31 (81.6%)	38
East North Central—42	156 (45.7%)	185 (54.3%)	341
West South Central—19	55 (36.7%)	95 (63.3%)	150
East South Central—10	33 (37.1%)	56 (62.9%)	89
South Atlantic—26	151 (44.3%)	190 (55.7%)	341
Middle Atlantic—40	125 (36.7%)	216 (63.3%)	341
New England—11	29 (31.5%)	64 (68.5%)	92
Total—188	697 (40.0%)	1,044 (60.0%)	1741

χ^2^ = 20.7, df = 8, Cramer's V = 0.109, *P* < 0.01.

Resident personal interests were provided for 867 (54%) residents. The most popular personal interest was outdoor activities (747, 86.2%), followed by exercise (642, 74.0%), family (300, 34.6%), video games (172, 19.8%), cooking (147, 17.0%), and traveling (28, 3.2%). An additional 408 (47.6%) personal interests that did not fit into the above categories were reported.

At the time of study, 2979 (84.2%) male and 560 (15.8%) female orthopaedic surgery residents were involved. Significant differences existed in specialty interests between male and female orthopaedic residents with male residents more interested in arthroplasty, spine, and sports and female residents more interested in hand and pediatrics (Tables 2 and 3). Female residents were more likely to report cooking as a personal interest (χ^2^ = 11.9, Phi = 0.1, *P* < 0.01), but no significant differences were found in reported interest in exercise, family, outdoor activities, travel, or video games between male and female residents (Tables 4 and 5).

**Table 3:** Percentage of Residents in the Same/Different Census Region From Their Medical School to Undergraduate Institution Location

US Census Division, No. of Programs	Same Region, No. of Residents (%)	Different Region, No. of Residents (%)	Total, No.
Pacific—20	122 (46.6%)	140 (53.4%)	262
Mountain—8	53 (60.9%)	34 (39.1%)	87
West North Central—12	28 (73.7%)	10 (26.3%)	38
East North Central—42	208 (61.0%)	133 (39.0%)	341
West South Central—19	96 (64.0%)	54 (36.0%)	150
East South Central—10	59 (66.3%)	30 (33.7%)	89
South Atlantic—26	193 (56.6%)	148 (43.4%)	341
Middle Atlantic—40	153 (44.9%)	188 (55.1%)	341
New England—11	47 (51.1%)	45 (48.9%)	92
Total—188	959 (55.1%)	782 (44.9%)	1,741

χ^2^ = 43.6, df = 8, Cramer's V = 0.158, *P* < 0.001.

**Table 4:** Resident Career Interests by Gender

Career Interest	Male (%)	Female (%)	Total (%)	χ^2^-value	*P*
Arthroplasty	140 (26.8%)	8 (9.4%)	148 (24.4%)	12.015	0.001
Foot and ankle	21 (4.0%)	6 (7.2%)	27 (4.4%)	1.585	0.208
Hand	74 (14.2%)	23 (27.1%)	97 (16.0%)	9.035	0.003
Oncology	18 (3.4%)	5 (5.9%)	23 (3.8%)	1.188	0.276
Pediatrics	25 (4.8%)	10 (11.8%)	35 (5.8%)	6.546	0.011
Shoulder and elbow	61 (11.7%)	13 (15.3%)	74 (12.2%)	0.889	0.346
Spine	76 (14.6%)	3 (3.5%)	79 (13.0%)	7.855	0.005
Sports	123 (23.6%)	9 (10.6%)	132 (21.7%)	7.231	0.007
Trauma	111 (21.3%)	18 (21.2%)	129 (21.3%)	0.000	0.985
Undecided	151 (28.9%)	29 (34.1%)	180 (29.7%)	0.944	0.331
Total	800	124	924	—	—

χ^2^ = 43.6, df = 8, Cramer's V = 0.158, *P* < 0.001.

**Table 5:** Resident Personal Interests by Gender

Personal Interest	Male (%)	Female (%)	Total (%)	χ^2^-value	*P*
Cooking	108 (15.0%)	39 (26.7%)	147 (16.9%)	11.927	0.001
Exercise	530 (73.4%)	112 (76.7%)	642 (74.0%)	0.689	0.407
Family	253 (35.0%)	47 (32.2%)	300 (34.6%)	0.436	0.509
Outdoor activities	627 (86.8%)	120 (82.2%)	747 (86.1%)	2.189	0.139
Travel	141 (19.5%)	33 (22.6%)	174 (20.0%)	0.771	0.380
Video games	25 (3.5%)	3 (2.1%)	28 (3.2%)	0.716	0.398
Other	362 (50.8%)	85 (58.6%)	447 (52.1%)	2.975	0.085
Total	2,046	439	2,485	—	—

## Discussion

Although orthopaedic surgery residents commonly attend programs in the same geographic region as their medical school (46.2%) and university (40.0%), they are more likely to leave for a different region. Orthopaedic residents also were found to more frequently attend university and medical school in the same geographic regions (959, 55.1%). The 2019 NRMP results suggested that geographic location was the single most important factor for all specialties in determining their rank list. These results also demonstrated that geographic location was the most important factor for orthopaedic surgery applicants, along with perceived goodness of fit and program reputation.^6^

Past studies have primarily considered the geographic region of resident training in the context of the census-provided regions. The US Census Bureau geographic regions vary drastically in their total area and may not be a true representation of distance traveled. Furthermore, geographic regions cannot be extrapolated as an indicator of program culture. Using a specific mileage traveled for each resident based on area code allows for a more precise assessment of the geographic movement patterns of orthopaedic residents.

A recent study found that 25% of general surgery applicants matched at an institution in their home state and 75% of applicants matched at a program less than 640 miles from their medical school or undergraduate college.^16^ In addition, they found that only 15% matched at an institution located over 1000 miles away and a mere 4% matched over 2000 miles away. We found the average distance between an orthopaedic resident's program and medical school to be 666 miles and 779 miles from their undergraduate institution.

The question remains as to why residents are more likely to stay in their specific geographic region. We hypothesize that both applicant- and program-associated factors are likely involved, leading to this outcome. Although the NRMP prohibits inquiry by programs into the geographic preferences of applicants, selection biases might exist on both the part of the applicant and residency program. The evidence for selection biases based on geographic preference in orthopaedic surgery, however, is lacking. Many public institutions may have incentive to retain talent to practice locally in those geographic regions. Similarly, applicants are likely to have a geographic preference for various social, socioeconomic, and career-directed factors, despite whether they voluntarily disclose that information to interviewing programs. Certain regions also have less programs and therefore less opportunity to match and remain in the same region as their medical school. This factor may play a role in the migration of medical students to other areas of the country. Our data suggest that this factor does influence smaller regions such as the Mountain (8 programs), East South Central (10 programs), and New England (11 programs) regions, where less residency programs correlate with fewer residents remaining in the same region as their medical school. Conversely, the East North Central (42) and Mid-Atlantic (40) have the most programs and retain more medical students as residents (Figures 1 and 2).

This study secondarily examined the career and personal interests of orthopaedic surgery residents. Although the fellowship match is years away for orthopaedic applicants, less than 65% of residents obtain their first two choices in the fellowship match.^17^ A basic understanding of the fellowship interests of current residents may influence applicant decision making if they feel inclined toward a specific specialty. Our data follow the steadily increasing trend in interest for arthroplasty.^18^ Arthroplasty was found as the top-rated interest among current residents and sports, trauma, and hand subsequently followed. Our data also reflect published 2015 data, in which the most popular fellowship areas were sports and then hand and spine.^17^ Pediatrics has remained the top chosen fellowship among women in orthopaedics,^19^ and this study further supports this finding. In addition, female orthopaedic residents were markedly more likely to report hand as a career interest.

Personal interests are a unique data set to this study. One study found that applicants consider interactions with current residents to be the most important part of the interview process,^20^ and aligning interests with potential coresidents helps applicants to assess their fit at a given program. For example, according to the data in this study, 34.6% of residents listed family as one of their most important hobbies, and a prospective orthopaedic applicant who identified family as a personal interest is likely to judge their fit for a program through perception of that program's family friendliness. Standardization and availability of resident hobbies and life interests may provide prospective applicants with improved insights for best fit.

To the authors' knowledge, this is the first study to report the program-provided career and personal interests of orthopaedic surgery residents. Our analyses provide a four-year update of orthopaedic surgery resident geographic distribution trends compared with the lone previous study^11^; however, this study also includes data of both allopathic and osteopathic orthopaedic surgery programs, as well as a more precise determination of mean geographical distance traveled by each resident from university and medical school to residency.

This study must be considered in the context of its limitations. One major limitation of this study is that it relied on the data provided by orthopaedic residency program websites. Significant inconsistencies exist in data reporting between residency programs, and interdepartmental variability was also found where coding among residents was inconsistent. An additional limitation is the large amount of data absent from our data set due to these inconsistencies; however, this remains the largest study of its kind to date. Generalization of US Census Bureau geographic regions represents a further limitation of this study, but these regions were included for standardization purposes and are consistently used in similar studies. We offer a numeric calculation of geographic distance in the form of miles traveled to counter this potential limitation. Another limitation of this study is the high ratio of men to women in our sample (5.3:1). As women continue to be underrepresented in orthopaedic surgery,^21^ our study found that orthopaedic surgery residency programs are still training more than five times as many men as women, which may lead to the possibility of gender-influenced biases in our results.

## Conclusion

Orthopaedic surgery residents are more likely to train in a geographic region that is different from their medical school or undergraduate institution. However, residents are statistically more likely to remain in the same geographic region as their medical school and undergraduate institution than to leave for any other US census bureau region. The average distance between orthopaedic residents' medical school and residency program is 666 miles. Male residents were more interested in arthroplasty, spine, and sports, whereas female residents were more interested in hand and pediatrics. Personal interests seem to be similar between genders.

## References

[1] Bernstein AD Jazrawi LM Elbeshbeshy B Della Valle CJ Zuckerman JD: Orthopaedic resident-selection criteria. J Bone Joint Surg Am 2002;84:2090-2096. 12429773 10.2106/00004623-200211000-00026

[2] DePasse JM Palumbo MA Eberson CP Daniels AH: Academic characteristics of orthopaedic surgery residency applicants from 2007 to 2014. J Bone Joint Surg Am 2016;98:788-795. 27147692 10.2106/JBJS.15.00222

[3] Schenker ML Baldwin KD Israelite CL Levin LS Mehta S Ahn J: Selecting the best and brightest: A structured approach to orthopedic resident selection. J Surg Educ 2016;73:879-885. 27230568 10.1016/j.jsurg.2016.04.004

[4] Legato JM Fuller DA Kirbos C: Matching into an orthopedic residency: Which application components correlate with final rank list order?. J Surg Educ 2019;76:585-590. 30206034 10.1016/j.jsurg.2018.08.018

[5] Sanfilippo JA Sharkey PF Parvizi J: Criteria used by medical students to rank orthopedic surgery residency programs. Am J Orthop 2006;35:512-514. 17152972

[6] National Resident Matching Program, Data Release and Research Committee. Results of the 2019 NRMP Applicant Survey by Preferred Specialty and Applicant Type. Accessed April 16, 2020.

[7] Abzug JM Chafetz R Johanson NA Bosacco S Kleinbart F: Factors medical students use to select orthopedic surgery residency positions. Am J Orthop 2013;42:30-31. 23431537

[8] Johnson AP Svider PF Folbe AJ: An evaluation of geographic trends in the otolaryngology residency match: Home is where the heart is. JAMA Otolaryngol Head Neck Surg 2015;141:424-428. 25763804 10.1001/jamaoto.2015.0219

[9] Silvestre J Lin IC Serletti JM Chang B: Geographic trends in the plastic surgery match. J Surg Educ 2016;73:270-274. 26774936 10.1016/j.jsurg.2015.10.015

[10] Loh AR Joseph D Keenan JD Lietman TM Naseri A: Predictors of matching in an ophthalmology residency program. Ophthalmology 2013;120:865-870. 23260256 10.1016/j.ophtha.2012.09.028

[11] Cox RM Sobel AD Biercevicz A Eberson CP Mulcahey MK: Geographic trends in the orthopedic surgery residency match. J Grad Med Educ 2018;10:423-428. 30154974 10.4300/JGME-D-17-00633.1 PMC6108356

[12] Shappell CN Farnan JM McConville JF Martin SK: Geographic trends for United States allopathic seniors participating in the residency match: A descriptive analysis. J Gen Intern Med 2019;34:179-181. 30276657 10.1007/s11606-018-4686-1 PMC6374265

[13] Huntington WP Haines N Patt JC: What factors influence applicants' rankings of orthopaedic surgery residency programs in the National Resident Matching Program?. Clin Orthopaedics Relat Research® 2014;472:2859-2866. 10.1007/s11999-014-3692-9 PMC4117903 24898527

[14] O'Donnell SW Drolet BC Brower JP LaPorte D Eberson CP: Orthopaedic surgery residency: Perspectives of applicants and program directors on medical student away rotations. J Am Acad Orthopaedic Surgeons 2017;25:61-68. 10.5435/JAAOS-D-16-00099 28002215

[15] US Census Bureau: Geographic Terms and Concepts—Census Divisions and Census Regions. https://www.ncdc.noaa.gov/monitoring-references/maps/us-census-divisions.php. Accessed March 25, 2020.

[16] Dhar VK Hanseman DJ Young G: Does geographical bias impact the match for general surgery residents?. J Surg Educ 2020;77:260-266. 31677980 10.1016/j.jsurg.2019.09.018

[17] Cannada LK Luhmann SJ Hu SS Quinn RH: The fellowship match process: The history and a report of the current experience. J Bone Joint Surg Am 2015;97:e3. 25568401 10.2106/JBJS.M.01251

[18] Ruddell JH Eltorai AEM DePasse JM: Trends in the orthopaedic surgery subspecialty fellowship match: Assessment of 2010 to 2017 applicant and program data. J Bone Joint Surg Am 2018;100:e139. 30399086 10.2106/JBJS.18.00323

[19] Klyce W Nhan DT Dunham AM Dafrawy MHE Shannon C LaPorte DM: The times, they are A-changing: Women entering academic orthopedics today are choosing nonpediatric fellowships at a growing rate. J Surg Educ 2020;77:564–7. 31932218 10.1016/j.jsurg.2019.12.007

[20] Ramkumar PN Navarro SM Chughtai M Haeberle HS Taylor SA Mont MA: The orthopaedic surgery residency application process: An analysis of the applicant experience. J Am Acad Orthop Surg 2018;26:537-544. 29870416 10.5435/JAAOS-D-16-00835

[21] Poon Selina: Academic metrics do not explain the underrepresentation of women in orthopaedic training programs. JBJS 2019;101:e32. 10.2106/JBJS.17.01372 30994596

